# Prevalence, Patterns, and Risk Factors of Work-Related Musculoskeletal Diseases Among Teachers in the Eastern Province, Saudi Arabia: A Community-Based Retrospective Cross-Sectional Survey

**DOI:** 10.7759/cureus.32178

**Published:** 2022-12-04

**Authors:** Maitha K AlMaghlouth, Nasser M Alserhani, Farha A Aldossary, Munirah A Alabdulqader, Bassmh A Al-Dhafer

**Affiliations:** 1 College of Medicine, King Faisal University, Al Ahsa, SAU; 2 Orthopaedics, King Faisal University, Al Ahsa, SAU

**Keywords:** pain, saudi arabia, eastern province, work-related musculoskeletal diseases, teachers

## Abstract

Background and objective

Work-related musculoskeletal diseases (WMSDs) are one of the most common occupational health concerns among teachers. They refer to a variety of degenerative and inflammatory disorders that can be triggered by the work environment of the people affected. Teachers’ health is substantially significant as it impacts their productivity at work. The aim of this study was to determine the prevalence, patterns, implications, and risk factors of WMSDs among teachers in the Eastern Province of Saudi Arabia.

Methods

This study involved a survey based on a cross-sectional questionnaire that was distributed to 404 random school teachers located in the Eastern province of Saudi Arabia. Information such as sociodemographic characteristics, workplace characteristics, characteristics of WMSDs experienced, associated factors, and impact of the symptoms experienced was collected. Questions related to symptoms were obtained from the Nordic Musculoskeletal Questionnaire and Örebro Musculoskeletal Pain Screening Questionnaire (ÖMPSQ). The collected data were then analyzed using the SPSS Statistics version 23.0 (IBM Corp., Armonk, NY).

Results

Among the participants, back pain, shoulder pain, and knee pain were the most reported symptoms of WMSDs in the past 12 months, while elbow pain was the least experienced; 41.1% had pain, discomfort, or numbness that prevented them from performing regular daily activities. The regression analysis revealed a relationship between moderate to severe pain and a pain duration of more than 12 months (p=0.001). Additionally, those who were able to perform lighter work and were not having depression had a low probability of pain persistence and were able to carry out their usual work while experiencing pain (p=0.002).

Conclusions

In the Eastern province of Saudi Arabia, WMSDs were commonly reported by school teachers. The prevalence of WMSDs among teachers was 41.1%. The pain was described as disabling and causing a negative impact on performance. The most common locations of symptoms were the back, shoulder, and knees. Risk factors for severe pain were female gender, sleeping for six to seven hours, and pain located in the neck shoulder, elbow, hand, thigh/hips, back, knees, and ankles. Furthermore, severe pain was associated with pain duration of more than 12 months, affecting sleep, causing anxiety, and mild depression. The impact of severe pain seriously affected the daily activities of teachers.

## Introduction

Work-related musculoskeletal diseases (WMSDs) encompass a wide range of inflammatory and degenerative conditions that affect the muscles, ligaments, tendons, nerves, bones, and joints among people who engage in a variety of occupations, and can be caused by a single incident of or cumulative trauma [1]. WMSDs have been recognized as primary factors contributing to physical impairment, early retirement, and work-related absences and limitations, with an associated peak observed in the most productive period (middle age) in life, and can be financially burdensome for the individual, community, family, and the government [2].

WMSDs are the second major cause of disability globally, impacting almost all types of workers [3,4]. Among these affected working sectors is the teaching sector. Teachers perform a demanding job that involves more than just the act of teaching; it also entails planning classes, grading assignments, and participating in school activities that are not necessarily performed in a favorable environment with a head-down position [5,6]. Due to these factors, WMSDs have been reported to impact 39-95% of teachers [5]. Pain often leads to other issues, including persistent fatigue, sleep disturbances, excessive need for rest, withdrawal from activities, weakening of immunological function, and mental disorders [7]. 

Various risk factors for WMSDs have been described in the literature [8]. They range from patient-related (older age, female gender, number of children in the family, smoking history, sleeping habits) to work-related (working environment, workload, and the type of school where the teacher is employed). However, the literature also contains some conflicting data/information regarding certain contributing risk factors. For example, while some studies suggest that working for a longer period increased the chance of having WMSDs, other studies have stated that new teachers are more prone to getting WMSDs. On the contrary, some factors were found to play a major role in limiting and reducing the impact of WMSDs, such as a satisfactory working environment and performing regular exercise [9-11].

A recent study in Kenya by Elias et al. [2] has reported that being female and having low supervisor support are risk factors for getting WMSDs. A study by de Souza et al. [12] has found that bad sleep quality is highly associated with WMSDs in teachers in Brazilian public schools. In a more recent study, Kraemer et al. [13] found that teachers at federal institutes were exposed to several ergonomic risk factors, the most common of which were prolonged sitting and standing, sharp edges on work surfaces, having to use a laptop touchpad rather than a mouse, and incorrect screen height.

The Örebro Musculoskeletal Pain Screening Questionnaire (ÖMPSQ), developed by Linton and Halldén, is one of the most widely used tools for screening pain [14]. ÖMPSQ was created to aid in the identification of people at risk of acquiring chronic pain by looking into their attitudes, beliefs, and behavior in reaction to pain [15]. Management is mainly focused on physiotherapy for prevention and treatment, along with short-term use of non-steroidal anti-inflammatory medications [8,16].

The literature investigating WMSDs in Saudi Arabia has identified that the prevalence of low back pain symptoms among teachers is 63.8% [13]. A previous study conducted among Saudi female secondary school teachers in Al Khobar City, Saudi Arabia, found a high prevalence of musculoskeletal pain disorders (79.17%) [11]. However, none of the studies in the literature have discussed patterns and risk factors related to this condition. In light of this, this study aims to determine the prevalence, patterns, and risk factors of WMSDs among teachers in Saudi Arabia.

## Materials and methods

Study design and setting

This was a community-based retrospective cross-sectional study conducted in the Eastern Province of Saudi Arabia between December 2021 and March 2022.

Ethical approval

Prior to conducting the survey, ethical approval was obtained from the Institutional Research Board and the Ethics Committee of King Faisal University in Al Ahsa city after fulfilling all the required ethical criteria (Research Number: KFU-REC-2021-DEC-EA000300).

Study size and population

The study population included teachers employed in Eastern Province’s schools, including both public and private sectors, with at least one year of experience. Teachers with physical disabilities, malignant tumors, inflammatory joint diseases, pregnant teachers, and those who had undergone orthopedic surgery or/and retired were excluded from the study.

**Figure 1:**
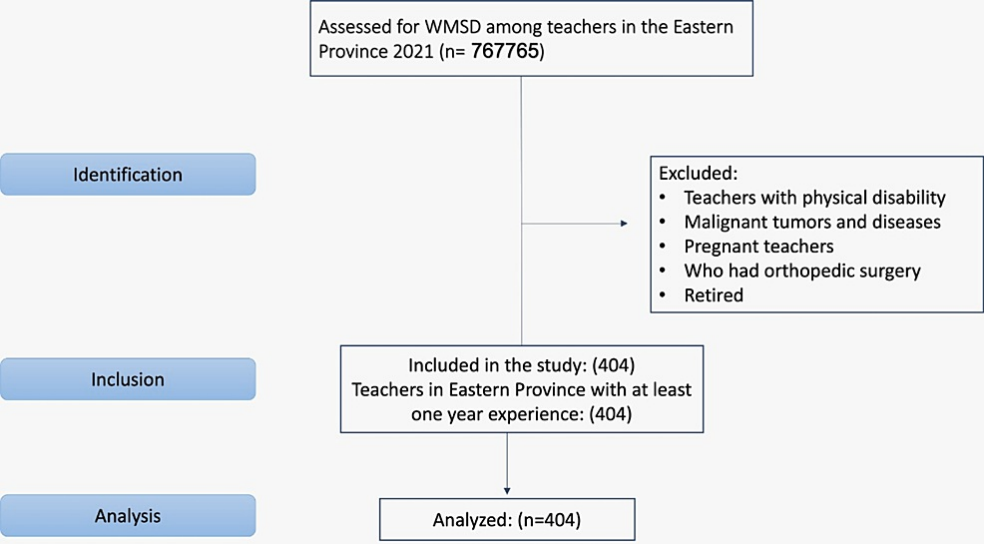
STROBE flow chart depicting the selection process STROBE: Strengthening the Reporting of Observational Studies in Epidemiology; WMSD: work-related musculoskeletal disease

According to the Ministry of Education, the total number of school teachers in the Eastern Region was 67,765 in 2021 [17]. Therefore, a sample of at least 377 teachers was required to participate in our study as calculated by Raosoft with a 95% level of confidence and a 5% margin of error [18]. We ultimately included 404 school teachers in our study. The Strengthening the Reporting of Observational Studies in Epidemiology (STROBE) flow chart illustrating the selection procedure is shown in Figure 1.

Data collection**

A survey was employed to collect data regarding the following aspects from participants; sociodemographics, workplace characteristics, characteristics of WMSDs experienced, associated factors, and impact of the symptoms experienced. Questions related to symptoms were obtained from the Nordic Musculoskeletal Questionnaire and ÖMPSQ [14,15]. 

The survey was conducted in Arabic, the primary language of the targeted population. The predesigned questionnaire was distributed among the community via a Google Forms link to enable better access to the population. Consent from the participants was taken prior to their filling out the form.

Statistical analysis 

The descriptive statistics of the collected data were presented using numbers and percentages. The relationship between the severity of pain and sociodemographic and other related pain characteristics of school teachers was assessed using the Chi-square test. A p-value <0.05 was considered statistically significant. All data analyses were performed using the SPSS Statistics version 23.0 (IBM Corp., Armonk, NY).

## Results

Demographics and habits 

A total of 404 school teachers participated in the study. The most common age group was 40-49 years (49.8%); more than half (52.2%) were female and nearly all (89.9%) were working in governmental institutions. Married participants constituted 84.7% of the sample. With respect to education, 90.3% were university or higher degree holders. Of note, 91.6% of the teachers earned more than 2,000 Saudi Riyals (SAR) per month. Nearly half of the teachers (49.3%) weighed between 70 and 89 kg, and 56.2% of the teachers reported having one to four children. Approximately 72.3% stated that the amount of sleep they get daily on average ranged from six to seven hours. In addition, a significant majority of the respondents (91.8%) were wearing medical shoes or shoes without heels (Table 1). 

**Table 1:** Sociodemographic characteristics of the school teachers (n=404)

Study variables	N (%)
Age group, years	
20–29	19 (4.7%)
30–39	84 (20.8%)
40–49	201 (49.8%)
50–59	100 (24.8%)
Gender	
Male	193 (47.8%)
Female	211 (52.2%)
Type of school	
Governmental	363 (89.9%)
Private	41 (10.1%)
Marital status	
Single	36 (8.9%)
Married	342 (84.7%)
Divorced	19 (4.7%)
Widowed	7 (1.7%)
Educational level	
Diploma or below	39 (9.7%)
University or above	365 (90.3%)
Monthly income, SAR	
≤2000	34 (8.4%)
>2000	370 (91.6%)
Weight group, kg	
30–49	6 (1.5%)
50–69	125 (30.9%)
70–89	199 (49.3%)
90–159	74 (18.3%)
Number of children	
None	57 (14.1%)
1–4	227 (56.2%)
5 or more	120 (29.7%)
Sleeping hours	
4–5	81 (20.0%)
6–7	292 (72.3%)
8–15	31 (7.7%)
Type of shoes	
Medical shoes or without heels	371 (91.8%)
With heels or not medical	33 (8.2%)

Teaching load

Regarding the workplace characteristics of participants, half of the teachers (50%) had 20-30 years of teaching experience with approximately 60.9% of them teaching one to four classes per day or 10-19 classes per week (46.3%), with a total workload of three to six hours per day of classes (51.5%). Also, 62.4% indicated having 20-34 students per class (Table 2).

**Table 2:** Workplace characteristics of teachers (n=404)

Variables	N (%)
Teaching experience, years	
1–9	78 (19.3%)
10–19	124 (30.7%)
20–30	202 (50.0%)
Classes per day	
1–4	246 (60.9%)
5–7	158 (39.1%)
Classes per week	
1–9	34 (8.4%)
10–19	187 (46.3%)
20–30	183 (45.3%)
Daily hours	
3–6	208 (51.5%)
7–9	196 (48.5%)
Number of students per class	
5–19	73 (18.1%)
20–34	252 (62.4%)
35–50	79 (19.6%)

Location of WMSDs experienced

**Figure 2:**
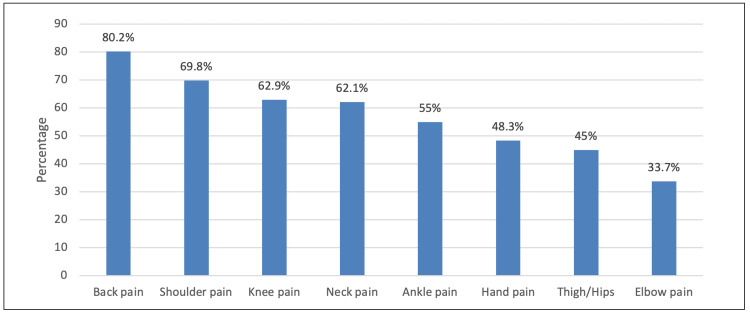
Prevalence of musculoskeletal pain in the past 12 months

**Figure 3:**
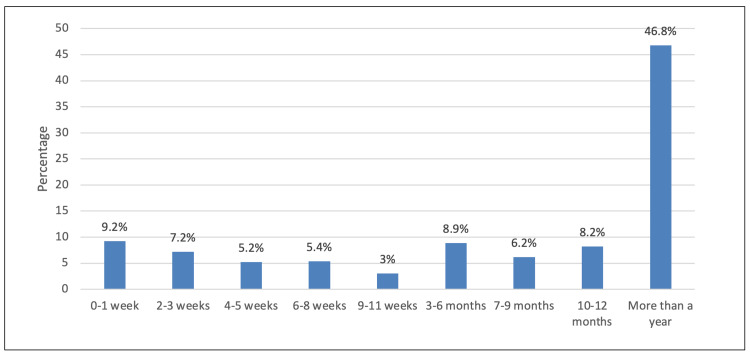
Duration of musculoskeletal pain, discomfort, or numbness

The prevalence in terms of the location of the pain experienced by participants in the past 12 months was as follows - back pain: 80.2%, shoulder pain: 69.8%, knee pain: 62.9%, and elbow pain: 33.7% (Figure 2); in nearly half of the participants (46.8%), the pain, discomfort, or numbness lasted for more than a year (Figure 3).

The physical and psychological impact of the symptoms of WMSDs

The characteristics of school teachers in dealing with pain, discomfort, and numbness in terms of dealing with pain, discomfort, and numbness is presented in Table 3. Pain, discomfort, or numbness preventing carrying out daily activities for the past 12 months was observed in 41.1% of teachers with 45.8% rating their pain as moderate. Nearly two-thirds reported that they can still do light work for an hour and sleep at night with the pain because it is not severe (64.9% and 60.9%, respectively). Additionally, the severity of the pain did not affect the ability to sleep at night. The proportion of teachers who were stressed or anxious for a week prior to filling out the questionnaire was 54.5% while that of teachers who felt severely depressed was 7.9%. Approximately 53.2% of the teachers believed that there is a low probability that the current pain will persist while 55.2% believed that there is a high probability that they can carry out daily activities normally for the next three months. Among the participants, 52.5% agreed that whenever the pain increases, they should stop what they usually do in order to reduce the pain, and 32.4% agreed that they should not be doing their usual work with their current pain (Table 3).

**Table 3:** Characteristics of school teachers in terms of dealing with pain, discomfort, and numbness (n=404)

Variables	N (%)
Characteristics of pain	
I had no pain or discomfort	95 (23.5%)
I have had pain, discomfort, and numbness in the past 7 days	143 (35.4%)
Pain, discomfort, or numbness has prevented me from carrying out normal daily activities for the past 12 months	166 (41.1%)
How would you rate the pain you've experienced over the past week?	
There is no pain	41 (10.1%)
Mild pain	141 (34.9%)
Moderate pain	185 (45.8%)
Severe pain	37 (9.2%)
I can do light work for an hour	
I can without pain	108 (26.7%)
I can with the pain because it is not severe	262 (64.9%)
I can't because of the pain	34 (8.4%)
I can sleep at night	
I can without pain	130 (32.2%)
I can with the pain because it is not severe	246 (60.9%)
I can't because of the pain	28 (6.9%)
How stressed or anxious have you felt in the past week?	
I feel calm and relaxed; no anxiety or stress	184 (45.5%)
I feel stressed or anxious	220 (54.5%)
How upset have you been feeling depressed over the past week?	
I didn't feel at all	216 (53.5%)
I felt depressed but not severely	156 (38.6%)
I felt severely depressed	32 (7.9%)
In your opinion, how much risk is there for your current pain to persist?	
There is no probability	154 (38.1%)
There is a low probability	215 (53.2%)
There is a high probability	35 (8.7%)
What is the probability that you will carry out your daily activities normally in the next 3 months?	
There is no probability	22 (5.4%)
There is a low probability	159 (39.4%)
There is a high probability	223 (55.2%)
If the pain increases, it is a sign that I should stop doing what I am doing until the pain subsides	
Agree	212 (52.5%)
Neutral	136 (33.7%)
Disagree	56 (13.9%)
I shouldn't be doing my usual work with my current pain	
Agree	131 (32.4%)
Neutral	177 (43.8%)
Disagree	96 (23.8%)

Risk factors for severe pain

We used the Chi-square test to determine the influence of pain severity on the sociodemographic characteristics of the teachers. The analysis revealed that the prevalence of moderate-severe pain was significantly more common among females (p<0.001), those who slept for six to seven hours, (p=0.036), and those who experienced musculoskeletal pain in the neck (p<0.001), shoulder (p<0.001), elbow (p<0.001), hand (p<0.001), thigh/hips (p<0.001), back (p<0.001), knees (p<0.001) and ankles (p<0.001) (Table 4).

**Table 4:** Factors that influenced pain severity (n=404) ^§^P-value has been calculated using the Chi-square test; *variables with multiple responses; **significant at p<0.05 level

Factor	Severity of pain		P-value^§^
	Mild/no pain, n (%) (n=182)	Moderate/severe pain, n (%) (n=222)	
Age group, years			
<40	55 (30.2%)	48 (21.6%)	0.137
40–49	86 (47.3%)	115 (51.8%)	
50–59	41 (22.5%)	59 (26.6%)	
Gender			
Male	115 (63.2%)	78 (35.1%)	<0.001**
Female	67 (36.8%)	144 (64.9%)	
Weight group, kg			
<70	56 (30.8%)	75 (33.8%)	0.337
70–89	87 (47.8%)	112 (50.5%)	
90–159	39 (21.4%)	35 (15.8%)	
Number of children			
None	33 (18.1%)	24 (10.8%)	0.103
1–4	99 (54.4%)	128 (57.7%)	
5 or more	50 (27.5%)	70 (31.5%)	
Sleeping hours			
4–5	29 (15.9%)	52 (23.4%)	0.036**
6–7	143 (78.6%)	149 (67.1%)	
8–15	10 (5.5%)	21 (9.5%)	
Type of shoes			
Medical shoes or without heels	167 (91.8%)	204 (91.9%)	0.961
With heels or not medical	15 (8.2%)	18 (8.1%)	
Teaching experience, years			
1–9	42 (23.1%)	36 (16.2%)	0.212
10–19	52 (28.6%)	72 (32.4%)	
20–30	88 (48.4%)	114 (51.4%)	
Classes per week			
1–9	16 (8.8%)	18 (8.1%)	0.672
10–19	88 (48.4%)	99 (44.6%)	
20–30	78 (42.9%)	105 (47.3%)	
Daily hours			
3–6	103 (56.6%)	105 (47.3%)	0.063
7–9	79 (43.4%)	117 (52.7%)	
Musculoskeletal pain*			
Neck	87 (52.2%)	164 (73.9%)	<0.001**
Shoulder	102 (56.0%)	180 (81.1%)	<0.001**
Elbow	38 (20.9%)	98 (44.1%)	<0.001**
Hand	63 (34.6%)	132 (59.5%)	<0.001**
Back	122 (67.0%)	202 (91.0%)	<0.001**
Thigh/hips	56 (30.8%)	126 (56.8%)	<0.001**
Knees	85 (46.7%)	169 (76.1%)	<0.001**
Ankles	75 (41.2%)	147 (66.2%)	<0.001**

Factors associated with severe pain

Additionally, moderate to severe pain showed a statistically significant relationship with the following factors: having a duration of pain of more than 12 months (p<0.001), being able to perform light work because the pain is not severe (p<0.001), being able to sleep at night (p<0.001), feeling stress or anxiety in the past week (p<0.001), and feeling mildly depressed (p<0.001) (Table 5).

Perception of pain and its impact

When assessing the perception of those with moderate to severe pain, there was a statistically significant association with the expectation of pain persistence (p<0.001) as well as a perceived low probability of carrying out daily activity normally in the next three months (p<0.001). Finally, experiencing moderate to severe pain had a statistically significant relationship with teachers having neutral expectations as to whether they should or should not continue working while experiencing pain (p=0.002) (Table 5).

**Table 5:** Association between the severity of pain and the characteristics of school teachers in dealing with pain, discomfort, and numbness (n=404) ^§^P-value has been calculated using the Chi-square test; *significant at p<0.05 level

Factor	Severity of pain		P-value^§^
	Mild/no pain, n (%) (n=182)	Moderate/severe pain, n (%) (n=222)	
Duration of pain			
12 months or less	117 (64.3%)	98 (44.1%)	<0.001*
More than 12 months	65 (35.7%)	124 (55.9%)	
I can do light work for an hour			
I can without pain	87 (47.8%)	21 (09.5%)	<0.001*
I can with the pain because it is not severe	91 (50.0%)	171 (77.0%)	
I can't because of the pain	4 (2.2%)	30 (13.5%)	
I can sleep at night			
I can without pain	102 (56.0%)	28 (12.6%)	<0.001*
I can with the pain because it is not severe	79 (43.4%)	167 (75.2%)	
I can't because of the pain	1 (0.50%)	27 (12.2%)	
How stressed or anxious have you felt in the past week?			
I feel calm and relaxed; no anxiety or stress	113 (62.1%)	71 (32.0%)	<0.001*
I feel stressed or anxious	69 (37.9%)	151 (68.0%)	
How upset have you been feeling depressed over the past week?			
I didn't feel at all	120 (65.9%)	96 (43.2%)	<0.001*
I felt depressed but not severely	54 (29.7%)	102 (45.9%)	
I felt severely depressed	8 (4.4%)	24 (10.8%)	
In your opinion, how much risk is there for your current pain to persist?			
There is no probability	105 (57.7%)	49 (22.1%)	<0.001*
There is a low probability	73 (40.1%)	142 (64.0%)	
There is a high probability	4 (2.2%)	31 (14.0%)	
What is the probability that you will carry out your daily activities normally in the next 3 months?			
There is no probability	14 (7.7%)	8 (3.6%)	<0.001*
There is a low probability	42 (23.1%)	117 (52.7%)	
There is a high probability	126 (69.2%)	97 (43.7%)	
If the pain increases, it is a sign that I should stop doing what I am doing until the pain subsides			
Agree	93 (51.1%)	119 (53.6%)	0.242
Neutral	58 (31.9%)	78 (35.1%)	
Disagree	31 (17.0%)	25 (11.3%)	
I shouldn't be doing my usual work with my current pain			
Agree	50 (27.5%)	81 (36.5%)	0.002*
Neutral	74 (40.7%)	103 (46.4%)	
Disagree	58 (31.9%)	38 (17.1%)	

## Discussion

The main purpose of the study was to determine the prevalence of WMSDs among school teachers. We also sought to assess the risk factors for pain and their impact on the study participants. There have been 28 previous studies investigating WMSDs among teachers. The survey demonstrated that among 404 participants, back pain was the most reported WMSD (76.5%), and the highest number of participants (49.8%) was in the age group of 40-49 years. A study conducted in Qassim has reported the highest prevalence of WMSDs in the age group of 40 years and above (60.2%), and in Hail, the age group of 41-45 years accounted for 24.4% [4,16]. Moreover, back pain was the most common complaint, as reported by the Qassim study (68.8%) and the study from Hail (74.4%) [4,16]. These findings are in accordance with the previously reported literature from other countries, such as Sweden (72.5%), Brazil (95.1%), and Japan (62.7%) [19-22]. Of note, the study findings correlate with other papers in terms of pain being more prevalent in certain age groups, as teachers who work for a long time may develop WMSDs compared to their counterparts with less experience.

However, our finding that the shoulders (69.8%) and knees (62.9%) constituted the second and the third most affected regions contrasted with other previously published studies in Brazil, North Ethiopia, and other developing countries, which indicated the neck as the second most affected [6-10,13], and with studies in Hong Kong, Brazil, and Sweden where they reported upper limbs as a third most affected region [4,19,21-26]. These differences can be attributed to differences between countries/regions in certain specific aspects related to the profession and workload itself. In this survey, most of the teachers stated that they have to stand for long hours while teaching, which puts high pressure on the lower back, shoulders, and knees. Meanwhile, in Brazil, North Ethiopia, and other developing countries, most participants reported that they spend more hours in a sitting position with their necks flexed and their upper limbs not supported, which causes pain in those regions. 

Among the participants, 52.2% of female teachers reported WMSDs, which aligns with several studies suggesting that WMSDs are more prevalent among women and are strongly related to the female gender [27]. The female gender has been found to be associated with different musculoskeletal disorders [28]. Regarding WMSDs, researchers have hypothesized that women are more likely to experience pain primarily due to their weaker physical capabilities, greater family obligations, and different career prospects compared to men; alternatively, men and women may simply report pain differently [21]. In a previous study, it was found that women experienced higher pain intensities, more disabilities, and greater difficulties in functioning than men. Furthermore, anxiety, depression, and self-efficacy among female teachers have been reported to be worse when compared to their male counterparts [3,29]. It has been reported that the above-mentioned differences are associated with several biochemical and biological characteristics [30].

Afferents C and Aẟ in male and female muscles are mechanically sensitive and respond to mechanical distortion, but females have a higher number of afferents and produce higher levels of cytokines as a result of tissue damage, which would indicate a stronger inflammatory response than their male counterparts [28,29].

In terms of the impact of WMSDs, about (12.2%) of teachers in our study complained of difficulty sleeping due to the severity of the pain. This finding demonstrated the huge impact WMSDs have on their lives. Similarly, a study conducted in Kenya showed that 52% of the participants had a decline in daily activities in the past 12 months due to low back pain. Half of those who experienced low back pain (51.5%) had suffered the pain in the past week [5]. In our study, 41.1% of teachers faced difficulties in carrying out their daily activities for the past 12 months and 54.5% of teachers were stressed or anxious in the week prior to filling out the questionnaire. This is consistent with a study conducted in North Ethiopia, which reported a comparable percentage of 60.9% [10]. The prevalence of teachers who felt depressed or severely depressed in our study was discovered to be 7.9% and 38.6%, respectively. Similar findings were observed in a study done in France that recognized major depressive disorder among male and female teachers to account for 23.2% and 40.8%, respectively [31]. Moreover, our study reported a probability of 75.8% of carrying out activities normally in three months, which is higher compared to the study done in Al Khobar city by 22.5% [11]. Such a finding can be explained by the fact that our study covered a larger geographical area. 

Based on the findings of this study, we strongly recommend that the concerned authorities take immediate actions to prevent the factors significantly associated with WMSDs in order to reduce the incidence of pain and associated disability. Changes in the types of schools, methods of teaching, and other variables may be difficult to bring about. As a result, educational programs for raising awareness of WMSDs and preventive methods such as regular exercise, as well as physiotherapy and medical treatment, should be implemented to alleviate pain in school teachers [8,32].

Limitations 

There are several limitations to our study that can be avoided in future research. Recall bias and self-reporting can be considered limitations of several studies of this nature given that many of these use anonymous surveys for data collection. Data were collected through online surveys due to constraints related to time, and this may have introduced certain participant-related biases to these questionnaires. Consequently, generalizing the results of this study should be done with caution. Moreover, it is not possible to separate cause from effect in a cross-sectional study design, resulting in weak evidence linking exposure and outcomes.

## Conclusions

The current study showed a high prevalence of work-related musculoskeletal pain disorders among teachers in the Eastern Region of Saudi Arabia. The majority of teachers regarded pain as disabling, which had a negative impact on their workplace attendance. Risk factors for severe pain were female gender, sleeping for six to seven hours, and pain located in the neck, shoulder, elbow, hand, thigh/hips, back, knees, and ankles. Furthermore, severe pain was associated with pain duration of more than 12 months, affecting sleep, causing anxiety, and mild depression. The impact of severe pain limited daily activities. To prevent this, teachers must be encouraged to participate in health-awareness programs. Regular exercise should be included in these programs, as it may be the most effective way to relieve pain and suffering, resulting in fewer WMSDs. More importantly, to reduce the risk of long-term disability, appropriate measures are required. These actions can be linked to changes in personal and environmental factors, which will help teachers improve their health. Further studies are needed to gain deeper insights into factors such as gender differences in the incidence and severity of WMSDs as well as WMSDs' association with the number of years in terms of teaching experience as a risk factor on a larger scale.

## Appendices

Appendix 1

**Table 6:** Musculoskeletal pain among school teachers - questionnaire

Dear participant, this study aims to assess and measure the extent of musculoskeletal pain among school teachers. This study targets school teachers in the Eastern Province of the Kingdom of Saudi Arabia. The data entered will be treated confidentially and only researchers will see it. Your participation in this study is voluntary, and you have the full right not to complete and return the questionnaire.
Biographical Data
Do you agree to participate in the questionnaire? Yes, No
Gender: Male, Female
Workplace: State school, Civil School
Age group, years: 20-29, 30-39, 40-49, 50-59
Social situation: Single, Married, Divorced, Widower
Educational level: Diploma; College or above
Standard of living: Less than 2000 riyals, More than 2000 riyals
Weight: 30-49 kg, 50-69 Kg, 70-89 Kg, 90-159 Kg
Number of children: 0, 1-4, More than 5
Sleeping hours: 4-5, 6-7, 8-15
The shoes you wear most often: Shoes with heels, Loafers
Number of years of teaching: 1-9, 10-19, 20-30
Number of classes per day: 1-4, 5-7
Number of classes per week: 1-9, 10-19, 20-30
Daily working hours: 3-6, 7-9
Number of students per class: 5-19, 20-34, 35-50
Scandinavian Musculoskeletal Questionnaire section
Have you experienced in the past 12 months (pain, discomfort, or numbness) in the neck? Yes, No
Have you experienced in the past 12 months (pain, discomfort, or numbness) in the shoulders? Yes, both shoulders, Yes, right shoulder, Yes, left shoulder, No
Have you experienced (pain, discomfort, or numbness) in the elbows during the past 12 months? Yes, both elbows, Yes, right elbow, Yes, left elbow, No
Have you experienced (pain, discomfort, or numbness) in the hands/wrists during the past 12 months? Yes, both wrists Yes, right wrist Yes, left wrist No
Have you experienced in the past 12 months (pain, discomfort, or numbness) in the back? Yes, full back, Yes, upper back, Yes, lower back, No
Have you experienced in the past 12 months (pain, discomfort, or numbness) in one or both thighs/hips? Yes, No
Have you experienced in the past 12 months (pain, discomfort, or numbness) in one or both knees? Yes, No
Have you experienced in the past 12 months (pain, discomfort, or numbness) in one or both feet/ankles? Yes, No
If you answered “yes” to one of the above questions, choose one of these sentences: You have experienced pain, discomfort, and numbness in the past 7 days, Pain, discomfort, or anesthesia has prevented me from carrying out normal daily activities for the past 12 months, I had no pain, discomfort, or sedation
Orebro Musculoskeletal Pain Screening Questionnaire (Short)
How long have you been in pain, discomfort, or numbness? One week, 1-2 weeks, 2-3 weeks, 3-4 weeks, 4-5 weeks, 6-8 weeks, 9-11 weeks, 3-6 months, 6-9 months, 9-12 months, more than a year
Pain severity assessment
How would you rate the pain you've experienced over the past week? No pain, Mild pain, Moderate pain, Severe pain
I can do light work for an hour: I can't because of the pain, I can with the pain because it is not severe, I can without pain
I can sleep at night: I can't because of the pain, I can with the pain because it is not severe, I can without pain
How stressed or anxious have you felt in the past week? I feel nervous or anxious. I feel calm and relaxed. No anxiety or stress
How upset have you been with feeling depressed over the past week? I felt severely depressed. I felt depressed but not severely. I didn’t feel at all
In your opinion, what is the risk of continuing your current pain? No danger. There is a little danger. There is a great danger
What is the probability that you will carry out your daily activities normally within the next 3 months? No chance. There is a small chance. There is a great opportunity
If the pain increases, it means that it is a sign that I should stop doing what I am doing until the pain decreases: Disagree, Neutral, I agree
I should not do my usual business with my current pain: Disagree, Neutral, I agree

## References

[1] Aldera MA, Alexander CM, McGregor AH (2020). Prevalence and incidence of low back pain in the Kingdom of Saudi Arabia: a systematic review. J Epidemiol Glob Health.

[2] Elias HE, Downing R, Mwangi A (2019). Low back pain among primary school teachers in Rural Kenya: prevalence and contributing factors. Afr J Prim Health Care Fam Med.

[3] Munala JM, Olivier B, Karuguti WM, Karanja SM (2021). Prevalence of musculoskeletal disorders amongst flower farm workers in Kenya. S Afr J Physiother.

[4] Cromie JE, Robertson VJ, Best MO (2000). Work-related musculoskeletal disorders in physical therapists: prevalence, severity, risks, and responses. Phys Ther.

[5] Althomali OW, Amin J, Alghamdi W, Shaik DH (2021). Prevalence and factors associated with musculoskeletal disorders among secondary schoolteachers in Hail, Saudi Arabia: a cross-sectional survey. Int J Environ Res Public Health.

[6] Chiu TT, Lam PK (2007). The prevalence of and risk factors for neck pain and upper limb pain among secondary school teachers in Hong Kong. J Occup Rehabil.

[7] Kerssens JJ, Verhaak PF, Bartelds AI, Sorbi MJ, Bensing JM (2002). Unexplained severe chronic pain in general practice. Eur J Pain.

[8] Erick PN, Smith DR (2011). A systematic review of musculoskeletal disorders among school teachers. BMC Musculoskelet Disord.

[9] Erick PN, Smith DR (2015). Musculoskeletal disorders in the teaching profession: an emerging workplace hazard with significant repercussions for developing countries. Ind Health.

[10] Kebede A, Abebe SM, Woldie H, Yenit MK (2019). Low back pain and associated factors among primary school teachers in Mekele City, North Ethiopia: a cross-sectional study. Occup Ther Int.

[11] Darwish MA, Al-Zuhair SZ (2013). Musculoskeletal pain disorders among secondary school Saudi female teachers. Pain Res Treat.

[12] de Souza JM, Pinto RZ, Tebar WR (2020). Association of musculoskeletal pain with poor sleep quality in public school teachers. Work.

[13] Kraemer K, Moreira MF, Guimarães B (2021). Musculoskeletal pain and ergonomic risks in teachers of a federal institution. Rev Bras Med Trab.

[14] Linton SJ, Halldén K (1998). Can we screen for problematic back pain? A screening questionnaire for predicting outcome in acute and subacute back pain. Clin J Pain.

[15] Brown G (2008). The Orebro musculoskeletal pain questionnaire. Occup Med (Lond).

[16] Aldukhayel A, Almeathem FK, Aldughayyim AA, Almeshal RA, Almeshal EA, Alsaud JS, Albaltan RI (2021). Musculoskeletal pain among school teachers in Qassim, Saudi Arabia: Prevalence, pattern, and its risk factors. Cureus.

[17] (2022). Data and Statistics, Ministry of Education. https://moe.gov.sa/ar/knowledgecenter/dataandstats/Pages/infoandstats.aspx.

[18] Charan J, Biswas T (2013). How to calculate sample size for different study designs in medical research?. Indian J Psychol Med.

[19] Cardoso JP, Ribeiro ID, de Araújo TM, Carvalho FM, dos Reis EJ (2009). Prevalence of musculoskeletal pain in teachers (Article in Portuguese). Rev Bras Epidemiol.

[20] Yamamoto N, Saeki K, Kurumatani N (2003). Work-related musculoskeletal disorders and associated factors in teachers of physically and intellectually disabled pupils. J Nara Med Assoc.

[21] Fjellman-Wiklund A, Sundelin G (1998). Musculoskeletal discomfort of music teachers: an eight-year perspective and psychosocial work factors. Int J Occup Environ Health.

[22] Fjellman-Wiklund A, Brulin C, Sundelin G (2003). Physical and psychosocial work-related risk factors associated with neck-shoulder discomfort in male and female music teachers. Med Probl Perform Art.

[23] Chong EY, Chan AH (2010). Subjective health complaints of teachers from primary and secondary schools in Hong Kong. Int J Occup Saf Ergon.

[24] Korkmaz NC, Cavlak U, Telci EA (2011). Musculoskeletal pain, associated risk factors and coping strategies in schoolteachers. Sci Res Essays.

[25] Atlas A, Bondoc RG, Garrovillas RA, Lo RD, Recinto J, Yu KJ (2007). Prevalence of low back pain among public high school teachers in the city of Manila. Philippine J Allied Health Sci.

[26] Edling CW, Fjellman-Wiklund A (2009). Musculoskeletal disorders and asymmetric playing postures of the upper extremity and back in music teachers: a pilot study. Med Probl Perform Art.

[27] Queme LF, Jankowski MP (2019). Sex differences and mechanisms of muscle pain. Curr Opin Physiol.

[28] Stubbs D, Krebs E, Bair M, Damush T, Wu J, Sutherland J, Kroenke K (2010). Sex differences in pain and pain-related disability among primary care patients with chronic musculoskeletal pain. Pain Med.

[29] Rhudy JL, DelVentura JL, Terry EL, Bartley EJ, Olech E, Palit S, Kerr KL (2013). Emotional modulation of pain and spinal nociception in fibromyalgia. Pain.

[30] Linaker CH, Walker-Bone K (2015). Shoulder disorders and occupation. Best Pract Res Clin Rheumatol.

[31] Kovess-Masféty V, Sevilla-Dedieu C, Rios-Seidel C, Nerrière E, Chan Chee C (2006). Do teachers have more health problems? Results from a French cross-sectional survey. BMC Public Health.

[32] Babatunde OO, Jordan JL, Van der Windt DA, Hill JC, Foster NE, Protheroe J (2017). Effective treatment options for musculoskeletal pain in primary care: A systematic overview of current evidence. PLoS One.

